# Vertical ground reaction force variables derived from Loadsol® insoles during overground walking are valid and reliable

**DOI:** 10.1371/journal.pone.0339481

**Published:** 2025-12-29

**Authors:** Dylan J. Mulligan, Clare E. Milner

**Affiliations:** Department of Physical Therapy and Rehabilitation Sciences, Drexel University, Philadelphia, Pennsylvania, United States of America; Pennsylvania State University Main Campus: The Pennsylvania State University - University Park Campus, UNITED STATES OF AMERICA

## Abstract

Force sensing insoles for measuring vertical ground reaction force (VGRF) have several advantages over laboratory-based force plates. Data can easily be collected in real-world environments and long duration trials with multiple steps are feasible. However, the sampling rate of insoles is lower and only vertical force is recorded. The reliability and validity of Loadsol® force sensing insoles have been evaluated for treadmill walking, but not overground walking. The purpose of the study was to determine criterion-related validity and test-retest reliability of Loadsol® compared to gold standard force plates. Twenty healthy young adults walked overground across force plates while wearing insoles for two blocks of five trials. Variables of interest were extracted from each trial and averaged across trials and participants. Intraclass correlation coefficients (ICCs) determined criterion-related validity for Loadsol® variables compared to force plate variables. ICCs also determined within session test-retest reliability for Loadsol® and force plates. Bland Altman plots were generated to assess bias and 95% limits of agreement. Additionally, standard error of measurements (SEMs) and minimum detectable differences (MDDs) were calculated. Excellent validity and minimal bias with Loadsol® was found for passive peak, active peak, instantaneous loading rate, impulse, and stance time, with good validity and an overestimation bias for average loading rate. Excellent test-retest reliability comparable to force plate reliability was also found for all variables. The SEMs and MDDs reported here help to inform researchers about whether the Loadsol® are suited to answering their research questions. By comparing the anticipated changes in a VGRF variable with the MDD of that variable, researchers can determine if Loadsol® are appropriate for the context of their study.

## Introduction

Force sensing insoles provide an opportunity to measure vertical ground reaction forces (VGRF) outside the gait laboratory. This has several advantages, such as enabling data collection outside the laboratory in more natural environments or in clinics, which may improve understanding of gait in these conditions [1–3]. Using force measuring insoles may also eliminate the need for study participants with mobility limitations to travel to the laboratory to participate in research. Force sensing insoles can also measure every step over longer duration continuous walking trials [1,3]. However, these insoles only measure VGRF perpendicular to the insole [1]. In contrast, force plates measure vertical, anterior-posterior, and medial-lateral ground reaction force. Additionally, force sensing insoles measure VGRF at a lower sampling rate than force plates [2]. Lower sampling rates are known to affect the accuracy of ground reaction measurements, particularly peaks [2]. Force Plate measurements of VGRF are the gold standard in the field due to their high sampling rates and high precision [2]. Therefore, it is important to compare VGRF measured by force sensing insoles to force plate measurements to understand insole capabilities and limitations.

A commercially available force sensing insole is the Loadsol® 1 by Novel Electronics Inc. (Pittsburgh, PA, USA) The validity and reliability of Loadsol® force sensing insoles during level walking have been investigated previously. Criterion-related validity of Loadsol® variables compared to force plates during level walking on a treadmill ranged from moderate to excellent according to established guidelines [1,4–6]. The Loadsol® also had moderate to excellent between-day reliability [1,4]. Notably, walking was on an instrumented treadmill in these prior studies. However, VGRF peaks measured during treadmill walking may be smaller than during overground walking [7–9], although differences are inconsistent [10]. Comparison of other VGRF variables between treadmill and overground walking have not been reported. Validity and reliability for the OpenGo force measuring insole by Moticon (Munich, Germany) during treadmill walking have also been evaluated [11,12]. Given these differences, findings from treadmill studies cannot simply be extended to the overground condition.

To fully characterize the psychometric properties of a measurement device, both validity and reliability must be determined. Criterion-related validity is the comparison of measurement made with the device being evaluated to measurements made by the accepted gold standard device, which is the criterion reference [6]. Validity is a continuous variable and standard thresholds for intraclass correlation coefficients (ICCs) are used to indicate that the device has sufficient validity [6]. Once acceptable validity has been established, the reliability of the device for repeated measurements should also be determined. Test-retest reliability is the consistency of outcome variables when the same experimental condition is repeated [6]. Since human study participants themselves may perform inconsistently day to day, reliability evaluations for data collected during the same session may provide insight into device reliability by keeping participant variation consistent. To aid the interpretation of reliability findings, which are also a continuous variable like validity, minimum detectable difference (MDD) can be calculated. The MDD, sometimes referred to as minimum detectable change (MDC), provides an indication of how great a difference between experimental conditions must occur to consider it a true difference greater than the measurement error of the device [6].

Therefore, the purpose of this study was to assess criterion-related validity and within session test-retest reliability of VGRF variables measured by the Loadsol® during overground walking. We hypothesized that when compared to force plate measurements, the VGRF peak forces, loading rates, impulse, and stance time from the Loadsol® would have excellent criterion-related validity and within-session reliability similar to force plates. Additionally, MDD for each variable of interest was evaluated.

## Methods

This study was approved by the Drexel University Institutional Review Board under protocol #2204009192. Participants provided written informed consent before participating. Healthy adults between the ages of 18 and 29 years were recruited from the campus and surrounding area. Recruitment for this study began July 25^th^,2022 and ended July 14^th^, 2024. As part of a larger study, participants with a body mass index (BMI) between 18.5 and 29.9 kgm^-2^ were included. Participants were excluded from the study if they reported any current injuries or pain, were unable to walk at least 10m unassisted, had gait limitations, used a cane or other assistive device, or reported they were pregnant. Twenty adults 22.2 ± 1.9 years of age, height 1.67 ± 0.06 m, body mass 66.6 ± 8.4 kg, BMI 23.7 ± 3.0 kgm^-2^ were enrolled and participated. An a priori power analysis using published guidelines determined the minimum sample size needed to determine validity and reliability using ICCs [13]. With alpha 0.05, beta 0.2, minimum acceptable reliability of 0.7, and anticipated reliability of 0.9, a minimum sample size of 18 participants was indicated.

At the start of the visit, following written informed consent, participant height and weight were recorded on a physician scale (Detecto, Webb City MO, USA) to confirm that they were with the BMI limits for the study. Participants wore laboratory provided sneakers (New Balance, Boston, MA) and socks. The appropriately sized pair of Loadsol® insoles (Novel Inc. Pittsburgh PA, USA) were placed in the sneakers worn by the participant. The Loadsol® were then calibrated to body weight per manufacturer instructions. Participants completed two blocks of five good overground walking trials at a self-selected pace over an approximately four-meter long path interspersed with other locomotor activities. Participants completed 4 trials as warmup prior to trials being recorded. The two blocks of testing were separated by an approximately 15-minute long rest period. A trial was accepted if each foot had full contact with an individual force plate. Walking velocity was monitored by wireless timing gates (Brower Timing Systems, Draper, UT) spaced four meters apart. VGRFs were recorded at 1000 Hz (AMTI, Watertown, MA). Concurrently, VGRF data were recorded by the Loadsol® sampling at 100 Hz.

**Fig 1:**
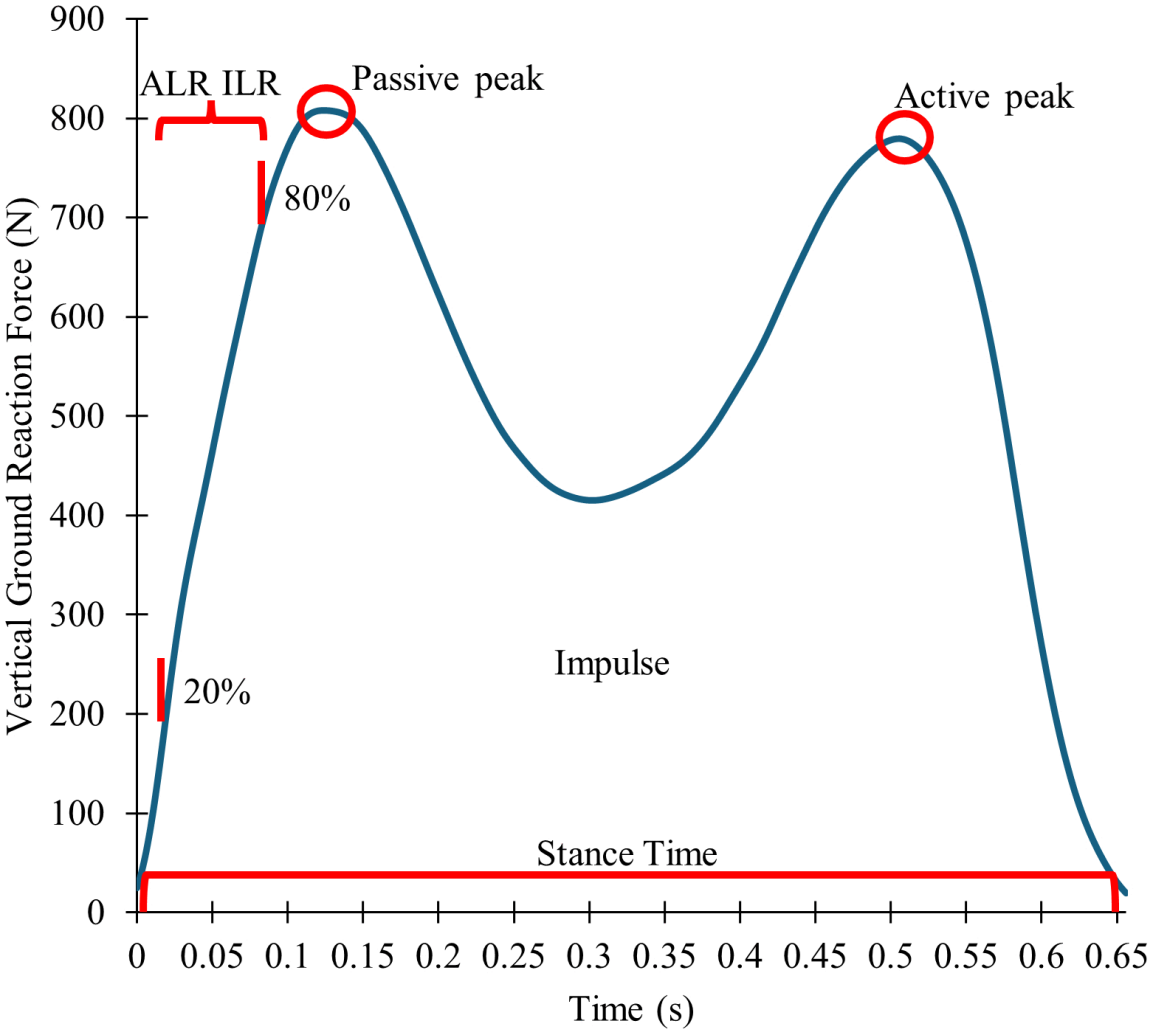
Definition of variables of interest in relation to a typical stance phase. Passive peak is represented by the red circle next to the label. Active peak is represented by the red circle next to its label. ALR, Average Loading Rate, and ILR, Instantaneous Loading Rate, are represented by the red bracket spanning from the 20% to 80% marks designating the area over which the variables are found. Impulse is represented by the area beneath the blue curve. Stance time is represented by the bracket showing the time occurring between foot contact and toe off.

Raw VGRF data from force plates and Loadsol® were imported into MATLAB (MathWorks, Natick, MA) for processing. Data were filtered using a fourth order low pass Butterworth filter with cutoffs of 30 Hz for force plate and 15 Hz for insole data. These cutoffs were determined from a residual analysis of pilot data from our laboratory. Stance phase was determined with a 20N threshold for VGRF to indicate foot strike and toe off. The variables of interest were extracted from the filtered data for right foot contacts in each trial to provide direct comparison between Loadsol and force plate data. The passive peak in VGRF was the first peak in stance phase and the active peak was the second peak in VGRF in the second half of stance were extracted (Fig 1). The average loading rate (ALR) was the mean slope of the VGRF between 20% and 80% of the time from foot contact to first peak. The instantaneous loading rate (ILR) was the highest loading rate between consecutive samples during the same time period. Impulse was the trapezoidal integration of the VGRF between foot strike and toe off. Stance time was the time between foot contact and toe off.

Variables were averaged across the five trials in each block for each device in each participant. Two-way mixed model ICCs (3,5) for absolute agreement were used to determine the validity of each variable for each device using SPSS software version 29. This ICC model was also used to determine the test-retest reliability of the insole variables in comparison to the gold standard force plate measures. Interpretation of reliability and validity was according to established thresholds. An *r* coefficient of at least 0.9 was excellent, good at least 0.75, and moderate at least 0.6 [6]. Additionally, standard error of measurement (SEM) and minimum detectable difference (MDD) were calculated for each variable [6].


$$SEM=SD\sqrt{\text{1}-ICC}$$

$$MDD=\text{1}.\text{9}\text{6}*SEM*\sqrt{\text{2}}$$

Bland Altman plots were used to assess the bias and 95% limits of agreement between the Loadsol® and the gold standard force plate [14].

## Results

**Fig 2:**
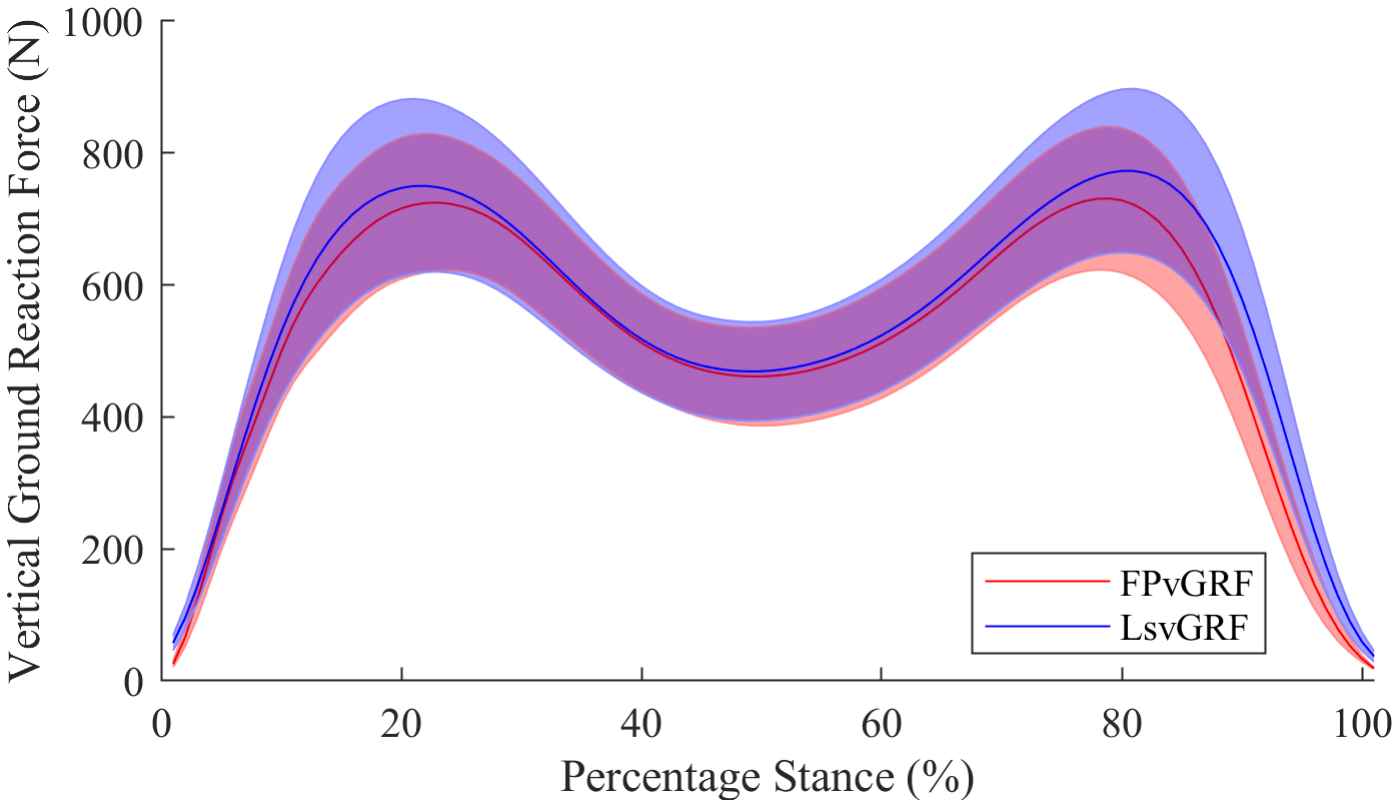
Ensemble average for vertical ground reaction force time series data for the stance phase of walking measured by Loadsol® force sensing insoles and force plates. The blue curve and shading represent the ensemble average from Loadsol® and the red curve and shading represents force plate data.

**Fig 3:**
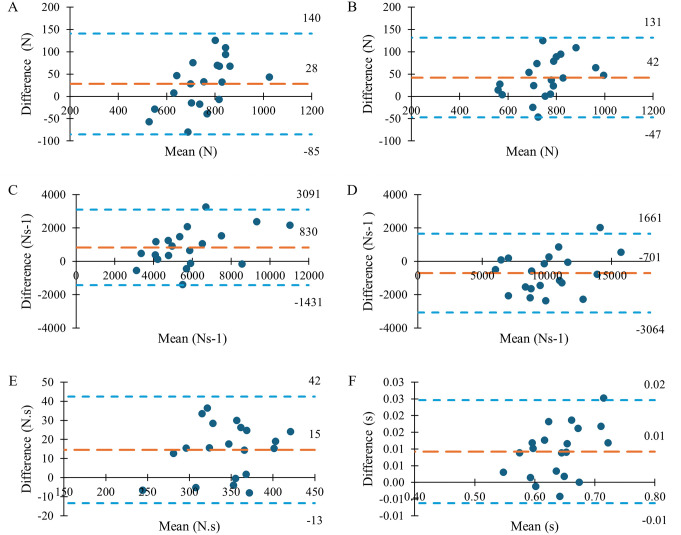
Bland Altman Plots showing 95% limits of agreement for all variables of interest. The orange long dashed lines represent the Loadsol® bias and the blue short dashed lines represent the 95% limit of agreement of the Loadsol®. A: Passive Peak, B: Active Peak, C: Average Loading Rate (ALR), D: Instantaneous Loading Rate (ILR), E: Impulse, F: Stance Time.

The average walking velocity was 1.43 ± 0.15 ms^-1^ in block 1 and 1.44 ± 0.14 ms^-1^ in block 2. The validity of the insole variables ranged from good for ALR to excellent for all other variables (Table 1; Fig 2). The test-retest reliability of VGRF variables determined from both insole (Table 2) and force plate (Table 3) data were excellent. The insole MDDs and SEMs were generally similar to those of the force plate. It should be noted that 95% confidence intervals for the validity of active peak, ALR, and impulse measured by the Loadsol® were wide. Bland-Altman plots indicated that the Loadsol® had a bias of −1% to 14% compared to the force plate (Fig 3).

**Table 1:** Criterion-related validity of vertical ground reaction force variables during overground walking measured using Loadsol® force sensing insoles compared to force plate measures.

	Device	Mean (SD)	ICC (95% CI)
**Passive peak (N)**	Force plate reference	739 (103)	0.930 (0.804–0.973)
	Loadsol®	766 (132)	
**Active peak (N)**	Force plate reference	736 (108)	0.933 (0.573–0.980)
	Loadsol®	778 (125)	
**Average Loading Rate (Ns**^**-1**^)	Force plate reference	5435 (1819)	0.886 (0.567–0.961)
	Loadsol®	6266 (2314)	
**Instantaneous loading rate (Ns**^**-1**^)	Force plate reference	10427 (2526)	0.936 (0.793–0.977)
	Loadsol®	9726 (2872)	
**Impulse (N.s)**	Force plate reference	337 (42)	0.948 (0.571–.986)
	Loadsol®	352 (45)	
**Stance Time (s)**	Force plate reference	0.64 (0.05)	0.984 (0.763–0.996)
	Loadsol®	0.63 (0.05)	

SD: standard deviation, ICC: intraclass correlation coefficient.

**Table 2:** Test-retest reliability of vertical ground reaction force variables during overground walking measured using Loadsol® force sensing insoles.

	Test mean (SD)	Retest mean (SD)	ICC (95% CI)	SEM	MDD
**Passive peak (N)**	766 (132)	769 (132)	0.988 (0.969–0.995)	15	40
**Active Peak (N)**	778 (125)	775 (126)	0.994 (0.985–0.998)	10	27
**Average Loading Rate (Ns**^**-1**^)	6266 (2314)	6308 (300)	0.969 (0.922–0.988)	406	1126
**Instantaneous loading rate (Ns**^**-1**^)	9726 (2872)	9976 (2754)	0.975 (0.938–0.990)	445	1233
**Impulse (N.s)**	352 (45)	352 (42)	0.987 (0.966–0.995)	5	14
**Stance Time (s)**	0.63 (0.05)	0.63 (0.05)	0.973 (0.966–0.989)	0.01	0.02

SD: standard deviation, ICC: intraclass correlation coefficient, SEM: standard error of measurement, MDD: minimum detectable difference.

**Table 3:** Test-retest reliability of vertical ground reaction force variables during overground walking measured using force plates.

	Test mean (SD)	Retest mean (SD)	ICC (95% CI)	SEM	MDD
**Passive peak (N)**	739 (103)	738 (100)	0.986(0.964–0.994)	12	33
**Active Peak (N)**	736 (108)	735 (104)	0.992 (0.980–0.997)	9	26
**Average Loading Rate (Ns**^**-1**^)	5435 (1819)	5433 (1877)	0.955 (0.886–0.982)	392	1087
**Instantaneous loading rate (Ns**^**-1**^)	10427 (2526)	10594 (2434)	0.942 (0.855–0.997)	597	1655
**Impulse (N.s)**	337 (42)	337 (38)	0.985 (0.963–0.994)	5	14
**Stance Time (s)**	0.64 (0.05)	0.64 (0.05)	0.972 (0.928–0.989)	0.01	0.02

SD: standard deviation, ICC: intraclass correlation coefficient, SEM: standard error of measurement, MDD: minimum detectable difference.

## Discussion

The purpose of this study was to determine the criterion-related validity and test-retest reliability of the Loadsol® force sensing insoles for determining VGRF variables during overground walking. Our hypothesis that the Loadsol® would have excellent criterion-related validity and within-session reliability similar to the force plates was supported for all variables, except validity of ALR which was good.

The validity of the Loadsol® in comparison to the force plate was generally excellent. The high validity of the passive peak (r = 0.93) in particular is supported by *r* values in the literature of 0.88 [5] and 0.89 [4] for treadmill walking. Similarly, a high *r* value (0.89) for VGRF peak (unspecified whether passive or active) was reported in another treadmill study [1]. While the previous series of studies chose not to filter the Loadsol data, we did apply a filter to remove high frequency noise from the signal. Presence of noise in the signal may affect the validity and reliability of the extracted data. Despite this difference in approach, prior findings are generally consistent with our results. Additionally, the bias of 4% is similar to the smaller than 5% bias previously reported for the Loadsol, and superior to the −35% for the OpenGo insole [4,5,12]. These findings support the use of Loadsol® for measuring passive VGRF peak during walking. The validity of ALR varied the most, with *r* values ranging from 0.72 [4] to 0.99 [5] and 0.889 in the present study. The ALR was overestimated by 14%, notably greater than previous reports of 2–3% for treadmill walking [4,5]. This variable is not a discrete point on the curve, but is the gradient of the curve during a portion of weight acceptance in early stance. Thus, it may be sensitive to the definition of foot contact and the region of interest over which it is calculated. If the definitions of ALR include different gradients, this may lead to increased differences between Loadsol® and force plate based measurements. Impulse had excellent validity in the present study (r = 0.948) and in the literature ranging from 0.95 to 0.96 [1,4,5]. The high validity of impulse across studies is likely because it is a summary measure and so less affected by any differences at individual timepoints. A small overestimation bias of 4% was found, this is in line with a previously reported bias of 0% for healthy young adults using the Loadsol® [4]. Despite similar methods, bias was larger in a study of Loadsol in older adults (15%) and with the OpenGo insole (−24%) [5,11]. Notably lower performance was reported with the OpenGo insole, likely due to a 50% lower sampling rate. Stance time also had excellent validity across the present and prior studies with bias of 1% or less [1,11]. Active peak and ILR have not previously been reported in the literature but were also found to have excellent validity in the present study. Collectively our findings and the supporting literature indicate that the Loadsol® accurately measures VGRF variables during walking.

The Loadsol® demonstrated excellent test-retest reliability for all VGRF variables. Similarly excellent within session test-retest reliability was reported previously for peak VGRF and impulse in healthy young adults during treadmill walking [1]. This pattern is also consistent for test-retest reliability between days [1,4]. Stance time also had excellent within day reliability in the present study and a previous study of treadmill walking [1]. The OpenGo insoles have also demonstrated excellent within day reliability for stance time [11]. Collectively, these findings demonstrate that the Loadsol® has excellent reliability during level walking both overground and on a treadmill. The active peak and ILR within session test-retest reliability have not previously been reported, though both had excellent reliability in the present study. Thus, test-retest reliability of the Loadsol® is excellent during both overground and treadmill walking.

The MDDs of VGRF variables have not been previously reported for Loadsol®. We found MDDs ranging from 3% of the mean value for stance time to 18% for ALR for the Loadsol®. The MDD values for the Loadsol® were comparable to those of the force plate which ranged from 3% for stance time to 20% for ALR. MDDs are based on the *r* value from the reliability ICC and the SEM and provide an indication of the magnitude of the measurement error in the variable of interest [6]. Thus, they are useful in guiding researchers on the minimum difference between groups or conditions that may be considered a meaningful difference larger than measurement error. This can be helpful in establishing whether the device is sensitive enough that the magnitude of important differences between conditions in a planned study could be detected. If the magnitude of important differences is less than or equal to the MDD the study analysis would not be able to determine if the differences were meaningful effects of experimental conditions or simply attributable to measurement error.

It should be noted that this study collected data for overground walking in young healthy adults. Therefore, these findings may not apply to other walking conditions such as incline or decline walking or to different participant populations. However, the purpose of this study was to determine validity and reliability of variables derived from Loadsol® compared to force plates. As such, if different study samples have force variables of similar magnitude to those reported in the present study, it may be expected that they will have similar psychometric properties for level walking. Furthermore, all data were collected using laboratory provided neutral running shoes. Standard footwear removes any variation in our findings that could be attributed to different shoe designs. While reliability wouldn’t be affected by shoe design, validity may be. For example, different force attenuation at the midsole (thicker of thinner midsole foam) may alter the relationship between force plate and Loadsol insole VGRF. This should be considered when interpreting the findings of this study in relation to other footwear.

## Conclusion

The results of this study indicate that the Loadsol® insole is both valid and reliable in measuring VGRF variables during overground walking with limited bias, while noting that validity was lower for ALR with a larger bias. Test-retest reliability was comparable to that of force plates. The SEMs and MDDs reported here help to inform researchers about whether the Loadsol® are suited to answering their research questions. By comparing the anticipated changes in a VGRF variable with the MDD of that variable, researchers can determine if Loadsol® are appropriate for the context of their study.

## Supporting information

S1 Table Participant average vertical ground reaction force variables for each block for force plate. (DOCX)

S2 Table Participant average vertical ground reaction force variables for each block for Loadsol®. (DOCX)
