# Supplementary material for: Vertical ground reaction force variables derived from Loadsol® insoles during overground walking are valid and reliable
Source: PLoS One. 2025 Dec 29;20(12):e0339481. doi: 10.1371/journal.pone.0339481 (PMC12747385; doi:10.1371/journal.pone.0339481)
Supplement: S1 Table — (DOCX) [file pone.0339481.s001.docx]

| **Participant #** | **Passive Peak 1 (N)** | **Active Peak 1 (N)** | **Average Loading Rate 1 (Ns^-1^)** | **Instantaneous Loading Rate 1 (Ns^-1^)** | **Impulse 1 (N*s)** | **Stance Time 1 (s)** | **Passive Peak 2 (N)** | **Active Peak 2 (N)** | **Average Loading Rate 2 (Ns^-1^)** | **Instantaneous Loading Rate 2 (Ns^-1^)** | **Impulse 2 (N*s)** | **Stance Time 1 (s)** |
| --- | --- | --- | --- | --- | --- | --- | --- | --- | --- | --- | --- | --- |
| 01 | 738 | 753 | 5073 | 10469 | 409 | 0.73 | 748 | 771 | 5608 | 10313 | 414 | 0.73 |
| 02 | 796 | 806 | 4706 | 9092 | 342 | 0.64 | 800 | 775 | 5113 | 9211 | 333 | 0.63 |
| 03 | 737 | 760 | 5532 | 11546 | 339 | 0.63 | 744 | 736 | 5543 | 11054 | 334 | 0.63 |
| 04 | 671 | 755 | 4121 | 10029 | 299 | 0.60 | 700 | 776 | 3770 | 9212 | 307 | 0.61 |
| 05 | 774 | 682 | 4488 | 9860 | 314 | 0.60 | 765 | 716 | 5557 | 9124 | 324 | 0.62 |
| 06 | 619 | 553 | 3532 | 6911 | 275 | 0.67 | 604 | 555 | 2962 | 7358 | 298 | 0.71 |
| 07 | 685 | 749 | 3889 | 9096 | 356 | 0.66 | 689 | 763 | 3620 | 8204 | 354 | 0.65 |
| 08 | 555 | 551 | 6192 | 9789 | 247 | 0.60 | 555 | 573 | 5079 | 11083 | 254 | 0.61 |
| 09 | 784 | 776 | 6737 | 11634 | 359 | 0.67 | 823 | 759 | 8038 | 12166 | 352 | 0.66 |
| 10 | 564 | 574 | 3388 | 6262 | 310 | 0.73 | 555 | 579 | 3588 | 7861 | 304 | 0.70 |
| 11 | 710 | 715 | 4586 | 9579 | 376 | 0.72 | 686 | 702 | 4438 | 10251 | 371 | 0.72 |
| 12 | 728 | 681 | 4141 | 8062 | 316 | 0.65 | 734 | 675 | 4590 | 7580 | 320 | 0.66 |
| 13 | 625 | 660 | 3128 | 6397 | 288 | 0.64 | 627 | 634 | 3312 | 7396 | 288 | 0.63 |
| 14 | 828 | 826 | 4610 | 13923 | 349 | 0.59 | 821 | 802 | 3978 | 11103 | 355 | 0.61 |
| 15 | 745 | 694 | 8642 | 11791 | 355 | 0.68 | 708 | 685 | 7726 | 12283 | 366 | 0.71 |
| 16 | 789 | 771 | 8136 | 13103 | 303 | 0.55 | 806 | 779 | 9700 | 13800 | 299 | 0.54 |
| 17 | 820 | 931 | 5960 | 11072 | 394 | 0.65 | 852 | 911 | 6644 | 13651 | 381 | 0.63 |
| 18 | 786 | 773 | 5913 | 10200 | 367 | 0.66 | 781 | 771 | 5295 | 10431 | 346 | 0.63 |
| 19 | 813 | 747 | 5987 | 14266 | 355 | 0.62 | 834 | 764 | 5340 | 15482 | 357 | 0.62 |
| 20 | 1002 | 972 | 9945 | 15462 | 394 | 0.58 | 933 | 979 | 8754 | 14312 | 382 | 0.57 |

**S1 Table: Participant average vertical ground reaction force variables for each block for force plate**
