# Supplementary material for: Vertical ground reaction force variables derived from Loadsol® insoles during overground walking are valid and reliable
Source: PLoS One. 2025 Dec 29;20(12):e0339481. doi: 10.1371/journal.pone.0339481 (PMC12747385; doi:10.1371/journal.pone.0339481)
Supplement: S2 Table — (DOCX) [file pone.0339481.s002.docx]

**S2 Table: Participant average vertical ground reaction force variables for each block for Loadsol**®

| **Participant #** | **Passive Peak 1 (N)** | **Active Peak 1 (N)** | **Average Loading Rate 1 (Ns^-1^)** | **Instantaneous Loading Rate 1 (Ns^-1^)** | **Impulse 1 (N*s)** | **Stance Time 1 (s)** | **Passive Peak 2 (N)** | **Active Peak 2 (N)** | **Average Loading Rate 2 (Ns^-1^)** | **Instantaneous Loading Rate 2 (Ns^-1^)** | **Impulse 2 (N*s)** | **Stance Time 1 (s)** |
| --- | --- | --- | --- | --- | --- | --- | --- | --- | --- | --- | --- | --- |
| 01 | 863 | 753 | 8328 | 11326 | 433 | 0.72 | 869 | 760 | 8105 | 12100 | 434 | 0.72 |
| 02 | 890 | 848 | 6778 | 8512 | 372 | 0.64 | 875 | 824 | 6580 | 7962 | 361 | 0.63 |
| 03 | 770 | 797 | 6175 | 10402 | 356 | 0.61 | 779 | 773 | 6131 | 10394 | 351 | 0.62 |
| 04 | 747 | 844 | 5375 | 10291 | 332 | 0.60 | 779 | 837 | 5496 | 9870 | 335 | 0.61 |
| 05 | 844 | 756 | 5403 | 9717 | 342 | 0.59 | 828 | 794 | 6609 | 9905 | 353 | 0.61 |
| 06 | 665 | 580 | 4715 | 7097 | 287 | 0.67 | 653 | 552 | 3631 | 7219 | 305 | 0.70 |
| 07 | 713 | 828 | 4281 | 7556 | 381 | 0.65 | 720 | 842 | 4365 | 7721 | 378 | 0.65 |
| 08 | 499 | 566 | 4804 | 7605 | 241 | 0.59 | 499 | 597 | 4034 | 8029 | 248 | 0.60 |
| 09 | 852 | 800 | 8264 | 11572 | 373 | 0.65 | 898 | 780 | 9367 | 11729 | 368 | 0.65 |
| 10 | 535 | 578 | 2843 | 5766 | 305 | 0.70 | 520 | 580 | 3258 | 6925 | 297 | 0.69 |
| 11 | 695 | 690 | 4936 | 7939 | 368 | 0.70 | 664 | 678 | 4442 | 7970 | 360 | 0.70 |
| 12 | 648 | 806 | 4259 | 5989 | 332 | 0.65 | 688 | 800 | 5199 | 6264 | 346 | 0.66 |
| 13 | 633 | 714 | 3595 | 6491 | 304 | 0.63 | 652 | 678 | 3866 | 7298 | 302 | 0.63 |
| 14 | 896 | 936 | 6090 | 11652 | 375 | 0.59 | 895 | 935 | 5363 | 10484 | 395 | 0.62 |
| 15 | 728 | 718 | 8478 | 10508 | 355 | 0.66 | 692 | 695 | 7901 | 10357 | 366 | 0.69 |
| 16 | 898 | 865 | 10512 | 15128 | 340 | 0.55 | 927 | 867 | 10976 | 15075 | 336 | 0.53 |
| 17 | 813 | 995 | 5841 | 8716 | 413 | 0.64 | 856 | 986 | 7010 | 11248 | 401 | 0.62 |
| 18 | 747 | 778 | 5475 | 8751 | 369 | 0.65 | 753 | 789 | 4820 | 8818 | 356 | 0.62 |
| 19 | 845 | 701 | 7050 | 13484 | 351 | 0.61 | 870 | 716 | 8375 | 15196 | 351 | 0.60 |
| 20 | 1045 | 1019 | 12112 | 16012 | 409 | 0.57 | 973 | 1026 | 10642 | 14963 | 396 | 0.55 |
